# Laboratory Performance Predicts the Success of Field Releases in Inbred Lines of the Egg Parasitoid *Trichogramma pretiosum* (Hymenoptera: Trichogrammatidae)

**DOI:** 10.1371/journal.pone.0146153

**Published:** 2016-01-05

**Authors:** Aloisio Coelho, Paul F. Rugman-Jones, Carolina Reigada, Richard Stouthamer, José R. P. Parra

**Affiliations:** 1 Escola Superior de Agricultura “Luiz de Queiroz”- ESALQ/ Universidade de São Paulo – USP, Piracicaba, SP, Brazil; 2 Department of Entomology, University of California, Riverside, California, United States of America; Natural Resources Canada, CANADA

## Abstract

In this study we assessed the relationship between the laboratory and field performance of different isofemale lines of *Trichogramma pretiosum* Riley. In comparative assays, we used three rare mitochondrial haplotypes as genetic markers of the isofemale lines, and by introgressing these mitochondrial haplotypes into each of 15 genetically different nuclear lines, also tested the assumption that mitochondria are neutral markers. In a laboratory trial, 45 isofemale lines (15 nuclear genotypes x three mitochondrial haplotypes) were ranked in three categories (best, intermediate and worst) according to the mean offspring production and the proportion of female offspring. Subsequently, lines from each of the three categories were selected for field releases to quantify field parasitism on *Ephestia kuehniella*. Temporally separate releases were done in a transgenic Bt cornfield, with four plots, each with 50 points of recapture. The points of recapture consisted of trap cards with eggs of *E*. *kuehniella* collected daily. The trap cards were maintained in the laboratory at 25°C until the adult wasps emerged, and the maternal identity of the wasps was determined using qPCR and high-resolution melt curve analysis to determine the mitochondrial haplotype. The results showed that these measures of laboratory performance (fecundity and offspring sex ratio) were good predictors of field success in *T*. *pretiosum*. We also report strong evidence discrediting the assumption that mitochondria are neutral, in view of the correlation between performance and mitochondrial haplotype.

## Introduction

The importance of genetics for biological-control applications has been a topic of much interest, with laboratory experiments demonstrating the general principles of population genetics. Indeed these principles apply to biological control agents [1, 2, 3], but little or no field work has been done to elucidate this aspect in released populations. In the process of biological control applications, several steps are involved that may influence the genetic makeup of (and variation within) a natural-enemy population. Almost all biological control operations involve three procedural steps: 1) Collecting the natural enemy from a wild population; 2) Maintaining the natural enemy in a laboratory (often a quarantine facility); 3) Mass rearing and release of the natural enemy in the field where they are needed to function as an inundative, augmentative or classical biological control agent [4, 5, 6, 7]. At each step, population genetic factors are thought to be important.

Haplo-diploid wasps such as *Trichogramma* spp. (Hymenoptera: Trichogrammatidae) undergo much lower levels of inbreeding depression than do diploid species [8, 9, 10, 11, 12, 13]. Even so, genetic adaptation to the laboratory will occur, and consequently the prediction is that with increasing effective population size during mass rearing, the performance of the parasitoid wasps should decrease under field conditions [14]. Some evidence exists that is consistent with adaptation of insects kept under laboratory conditions [14, 15, 16, 17], where initially it is very difficult to establish a population in the laboratory but after a number of generations their artificial rearing performance improved substantially. Experiments were carried out to determine the relationships of the fluctuating asymmetry of wing shape and size with the field performance of *Trichogramma carverae* (Oatman & Pinto, 1987) [18]. For this species, the laboratory fecundity as a predictor of field success was tested, as well [19]. In both cases no “direct competition” (release of parasitoids in the same experimental plot) between the experimental strains or population was arranged, because of the lack of a method to easily identify the strains.

For the improvement of biological control programs, studies are needed to assess the different intraspecific traits of the agents, especially in the field. Many behavioral traits are influenced by intraspecific genetic variation, and, better estimators of the ability of the biological control agent to respond to environmental conditions and increase its survival in the field are needed [20]. An efficient biological control agent is a natural enemy that is not only able to efficiently locate and attack a host, but that is also capable of remaining in a host-infested area until the host population is substantially reduced [7].

Species of *Trichogramma* are among the most-studied parasitoids worldwide [21, 22, 23]. In the 1980s at least 28 species were being released in 28 countries for the control of lepidopteran pests in annual and perennial crops [24, 25, 26] covering 32 million ha [27]. Yet, very few studies have compared the field success of different lines of the same species (but see [19]). One species, *T*. *pretiosum* Riley, is widely used as a commercial biological control agent in Brazil to control soybean pest in an area of 250,000 ha [28]. Considering the lack of information on the field performance of mass-reared *Trichogramma*, the present study attempted to determine the effect of the mass-rearing process by investigating the relationship between simple measures of laboratory performance and the subsequent field performance of inbred laboratory lines of *T*. *pretiosum*. For this purpose we used an innovative technique to “mark” different populations with unique mitochondrial haplotypes. Although these markers proved not be neutral as was expected, they did allow the field performance of *T*. *pretiosum* to be assessed in side-by-side releases.

## Material and Methods

### Isofemale lines

We used 15 from the 26 isofemale lines described by Gúzman-Larralde et al. in their experiment [29, 30] called as isoline 1, 6, 14, 26, 29, 35, 37, 38, 40, 42, 43, 46, 47, 51, 53, the protocol adopted by the authors was as follows. Eggs of the tobacco hornworm *Manduca sexta* (L.) (Lepidoptera: Sphingidae) were collected from tomato plants at the University of California’s South Coast Station, Irvine, in the summer of 2008. The weather in Irvine is classified as Mediterranean climate (Köppen Climate Classification: Csa) [31]. *T*. *pretiosum* is a tiny wasp (<1 mm) that parasitizes eggs of many insects, primarily Lepidoptera (18 genera in 9 families) [32]. Development is solitary or gregarious depending on host size. Oviposition to adult emergence requires 9–10 d at 24°C. The winged adults of both sexes disperse from the natal host patch within minutes or hours after emergence [33]. Average adult female longevity in the laboratory ranges from 1.6 to 7.0 d depending on host and honey availability [34]. Twenty-six single mated female wasp from each field-collected host egg was used to found an isofemale line, and reared under laboratory conditions, temperature 25 ± 1°C, RH 40 ± 10%, photophase 14h, using 24 h-old UV irradiated eggs of *Ephestia kuehniella* Zeller (Lepidoptera: Pyralidae) as factitious hosts. In each generation, a single female, <24 h old, was paired with her brother for 12 h to mate. This female was used to initiate the next generation. This inbreeding protocol was followed for nine generations. After nine generations, the resulting isofemale lines should have an inbreeding coefficient of at least 86% [35]. This inbreeding protocol will have removed most of the genetic variation within the lines, with less than 14% of the initial heterozygosity remaining within each line. At the start of our experiments, genetic differentiation across the 15 lines was demonstrated using two highly polymorphic microsatellite loci (nuclear DNA).

### Creation of replicate nuclear genetic lines in three mitochondrial backgrounds

**Fig 1:**
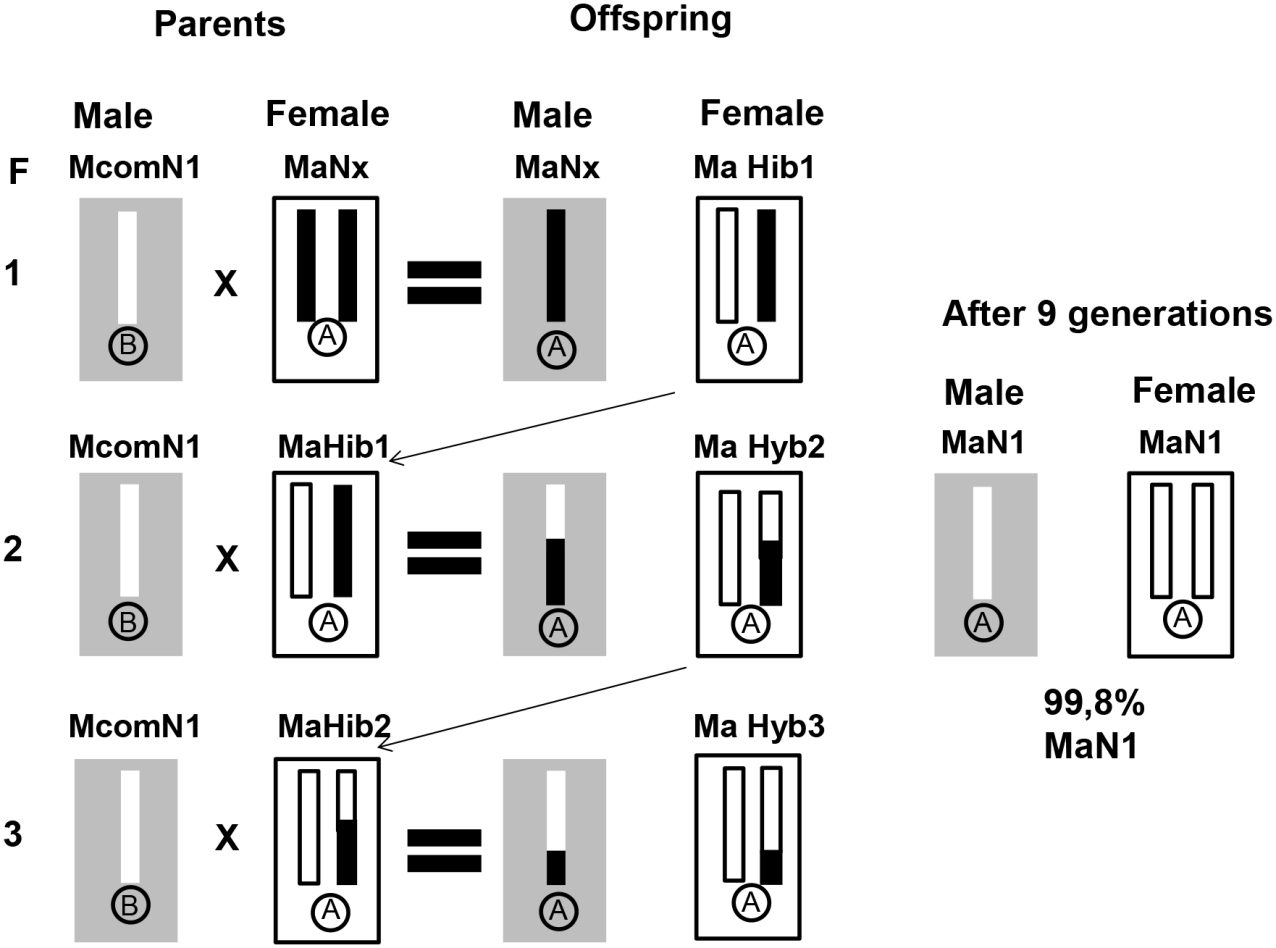
Crossing scheme to place the nuclear background N1 (The chromosome set is indicated by the white bar in the center of the gray rectangular boxes) into the mitochondrial background Ma (represented as A in a circle). In each generation “F” (indicated by the numbers), a single female offspring of the previous generation was mated with a male from the McomN1 line (giving rise to offspring with the genetic makeup shown to the right of the equals sign). With repeated backcrossing to males from the McomN1 line, an ever-increasing proportion of the nuclear genome of the backcrossed females (MaHyb) has the N1 type.

The mitochondrial background (haplotype) of each of the original field-collected *T*. *pretiosum* lines was determined by sequencing the barcoding region of the COI gene. Using with this information, three “rare” mitochondrial backgrounds (here referred to as Ma, Mb and Mc) that originated from Brazilian haplotypes of *T*. *pretiosum* haplotypes and could be distinguished from each other were selected and “placed” into each of the 15 inbred lines. Through a series of backcrosses, we created three replicates of each isofemale line that differed only in their mitochondrial marker (Fig 1). For example, we took an inbred line that initially had a mitochondrial background Mcom and a nuclear background N1 (McomN1), and through a series of backcrosses we created the following lines: MaN1, MbN1, McN1. To create the MaN1 line we first crossed males from the McomN1 line with females from a line that naturally “carried” Ma mitochondria, and then subsequently again crossed the female offspring resulting from this mating with the McomN1 males. This backcrossing protocol was repeated for 9 generations, after which the backcrossed line MaN1 was established. After nine generations of backcrossing, the MaN1 and McomN1lines will be, on average, (1-(0.5)^9^) = 0.998 identical in their nuclear genomic composition and essentially differ only in the mitochondrial haplotype that they harbor. At the end of this backcrossing exercise, we generated each of 15 nuclear backgrounds in three different mitochondrial backgrounds. A basic assumption of our experimental design was that mitochondria are neutral and do not affect the performance of the parasitoids (but see Results).

### High-resolution melt curves of the Real-Time PCR products (HRM)

**Fig 2:**
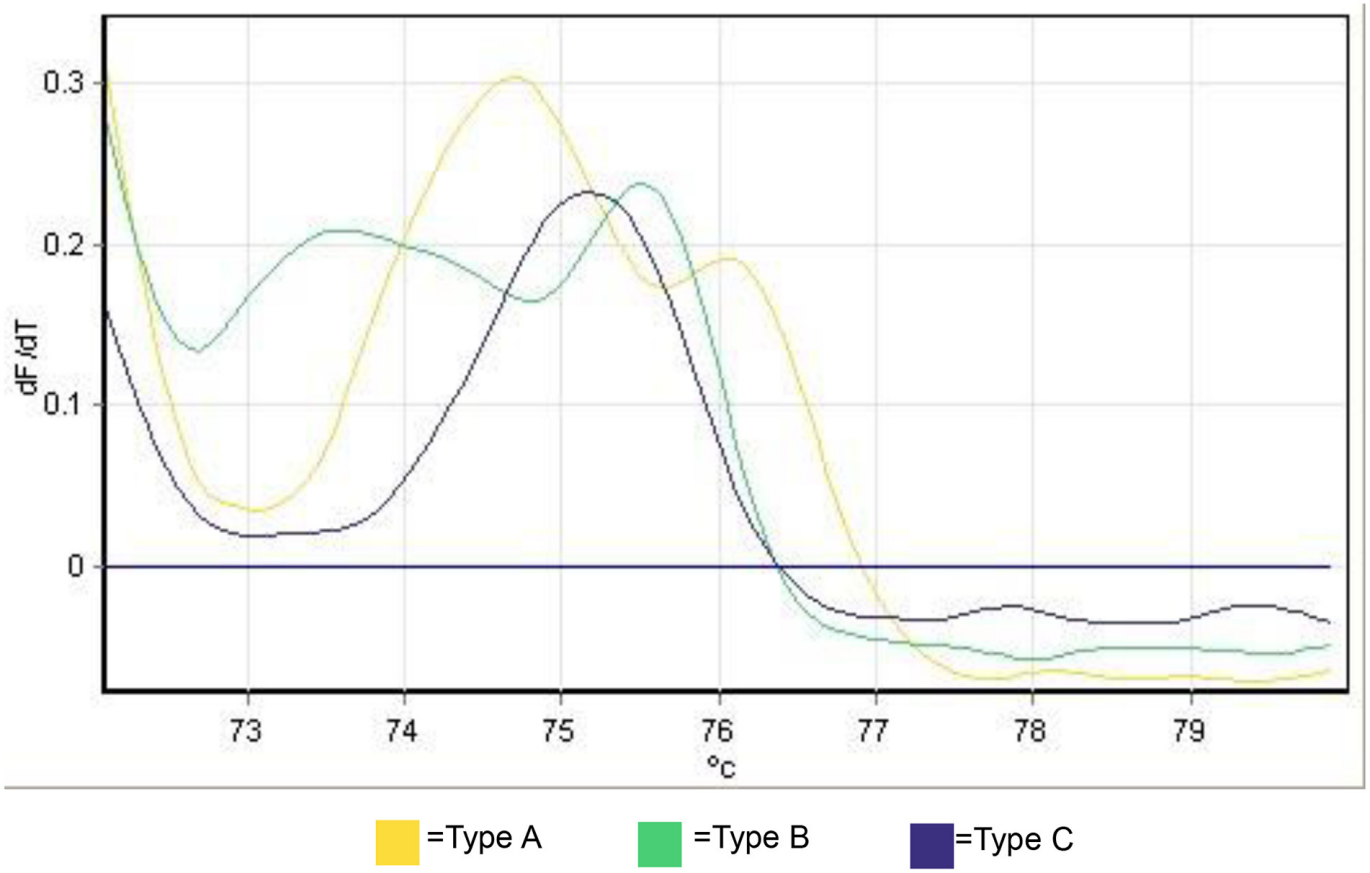
High-resolution melt curves for mitochondrial types A, B and C. Each type was consistently identifiable by the number and position of peaks. Height of peaks represents the relative fluorescence; the x axis indicates the temperature at the time that each reading was taken.

To easily identify the three experimental mitochondrial backgrounds, a high-resolution melt curve, based on a ~350 bp section of the COI gene, was developed. This method utilized whole wasps as a starting template, thereby avoiding the need for a separate DNA extraction, allowing the throughput required to complete the study. Reactions were carried out in 20 μl total volumes containing 400 nM uracil, 200 nM each adenine, guanine, and cytosine, 1 mM MgCl, 1X ThermoPol™ buffer (New England BioLabs), 0.25 μM forward primer (311F [this study]; 5’-TGGAACAGGTACAGGAACAGG-3’), 0.25 μM reverse primer (HCO2198 [36]; 5’-TAAACTTCAGGGTGACCAAAAAATCA-3’), 0.5X EvaGreen (Biotium), 1 U *Taq* polymerase (New England Biolabs), and 1 unit of Uracil-DNA Glycosylase (UDG). One whole individual *T*. *pretiosum* was added to each reaction tube. All reactions were done on Rotor-Gene RG-3000 (Corbett Research) or Rotor-Gene Q (Qiagen) instruments. Prior to amplification the reaction mixture was incubated for 10 min at 37°C, to allow the UDG to eliminate any potential carryover contamination [37]. Reaction mixtures were held at 95°C for 5 min, and cycled as follows: 95°C for 15 s, 55°C for 30 s, and 68°C for 30 s for 35 total cycles. Immediately following amplification, a melt analysis was conducted. PCR products were held at 72°C for 90 s and then the temperature was slowly increased in 0.2°C increments to a final temperature of 80°C. After each increase, the mixture was held for 10 s before the fluorescence was measured. The resulting melt curves showed distinctive patterns for mitochondrial types A, B and C (Fig 2).

### Laboratory ranking of the isofemale lines

Laboratory ranking of the isofemale lines was based on two measurements, fertility and offspring sex ratio. These measurements were evaluated for each of the 15 isofemale lines in each mitochondrial background (A, B, C), for a total of 45 treatments. From each isofemale line, 20 females (<24 h old) were placed in a Ø 1.5 X h 7 cm vial plugged with cotton. These females were fed with honey droplets and kept in a climate-controlled chamber at 25±1°C, with 60±10% relative humidity and a 14-h photophase. Each individual was offered a card containing approximately 125 *E*. *kuehniella* eggs. The egg card was replaced daily, and the parasitoid-exposed cards were maintained under the same climate conditions. The eggs were exposed for three days, and the parasitoid offspring were allowed to emerge from the host eggs. After emergence, the number of male and female offspring from each treatment (isofemale line X mitochondrial background) was counted to determine the fertility (measured as total offspring) and sex ratio (measured as proportion of females) of each line.

### Field experiment

Based on the prior assumption that we could use the mitochondria as a neutral marker that would not affect the performance of our experimental isofemale lines, we originally planned to conduct two separate field releases utilizing the same three nuclear lines, but each combined with a different mitochondrial marker in different releases. However, it became evident that the mitochondrion did in fact affect the performance (see Results). Therefore, as an alternative, two releases were conducted, each using a different set of three selected experimental lines (nuclear X mitochondrial background). The lines used were those that produced sufficient numbers of parasitoids for the releases: a “Best” line that produced many offspring with a high proportion of female offspring; a “Worst” line that produced relatively few offspring and a low proportion of female offspring; and an “Intermediate” line that performed between the two extremes. The field experiments were carried out in Piracicaba, São Paulo, Brazil (22° 70’ 01”S, 047° 64’ 31”W), using Bt corn plants (Dow^®^ 2B587PW/ pre-commercial product), to avoid natural infestation by lepidopterans. For each release, four plots of 10 x 10 m were prepared, 10 m apart. Each plot was delimited by flags placed at its corners. The size of the plots was defined based on the dispersal ability of *T*. *pretiosum* [38]. The corn plants in the plots were in vegetative stage V4/V5, i.e. plants with four or five complete leaves [39].

The release method used in these experiments followed the methods described by Kazmer and Luck [33]. Wasps were released from a center axis of the plots, using 50 ml Falcon^®^ tubes with honey streaked inside the tube. The lid of the tube was covered with a mesh that permits the parasitoids to exit while excluding predators. The tubes were placed in the field early in the morning in the interior rows of the plots. The number of host eggs in the vials was adjusted to produce approximately equal numbers of females, considering that 1 cm^2^ usually contained 500 *E*. *kuehniella* eggs. The cards were prepared considering the fertility and sex ratio of the isofemale lines. For the highly fertile isofemale lines, the parasitism was allowed for 6 h, and for the less-fertile lines the parasitism was allowed for 24 h. Aiming to release 1500 females per line in each plot, a host egg card 3.5 cm^2^ (containing approx. 1750 host eggs) was placed in the field. To better estimate the number of wasps released, the vials were collected after 24 h. Five 0.5 cm^2^ samples (70% of the released card) were taken from each egg card retrieved from the tubes, and the number of parasitized host eggs with exit holes was counted to determine the number of wasps that emerged per vial. Weather conditions during the release experiments were recorded by the weather station of the Biosystems Engineering Department of ESALQ/USP, located at the field site. The first release was on October 16 and the second on October 22, during the austral spring.

In each plot, 10 trap cards were hung (prepared using ~250 *E*. *kuehniella* eggs, 0.5 cm^2^ cards) at uniform distances apart, in each of five rows, for a total of 50 trap cards. Each row was 10 m long and the distance between rows was 1.90 m. The trap cards were replaced daily until the third day after the releases; after that time only a low percentage (less than 20%) of the trap cards would be expected to be parasitized by released wasps [33]. One day prior to each release, 20 trap cards were deployed in each plot to determine the activity of any endemic *Trichogramma*. All egg cards were kept in a climate-controlled cabinet, and the emerging specimens were stored in ethanol for subsequent molecular analysis to determine the identity of the mother. For each trap card collected from the field, the mitochondrial haplotype of a maximum of eight randomly sampled *T*. *pretiosum* individuals was determined using HRM analysis.

### Statistical analyses

The data from the laboratory ranking of the isofemale lines were analyzed using generalized linear models (GLM) [40]; the data for fertility through a quasi-poisson distribution, and the data for sex ratio by quasi-binomial distribution. The “F” value was calculated by factorial ANOVA of the models, considering as factors the nuclear background and the mitochondrial background (with interaction). Goodness-of-fit for all models was assessed using a half-normal graph of probabilities with a simulation envelope [41, 42]. When there was a significant difference between treatments, multiple comparisons (Tukey test, p< 0.05) were done by the glht function from the multcomp package [43] with “p” adjusted by single-step method, from the R statistical program (The R Foundation for Statistical Computing; http://www.R-project.org).

Parasitism rates in the field experiments were analyzed using modified Chi-square tests. Each trap card in the field could be parasitized by more than one female wasp. The number of females finding each card could not be directly determined, because we could not distinguish between an event in which one female of one mitochondrial type found a trap card and parasitized multiple eggs, and an event in which multiple females of the same type found and parasitized the same card. Therefore, rather than calculating a Chi-square value using a standard “observed” term, data from field experiments were analyzed using a modified Chi-square test (Dr. Leonard Nunney, personal communication). The modified “observed” term was found by subtracting the number of cards in each plot that were parasitized by a particular mitochondrial type from the total number of cards deployed in that plot. We then calculated the expected number of trap cards that were not found by each particular type, based on the numbers of female wasps of each type released and assuming that all mitochondrial types had the same chance (p) of not finding a trap card. We assumed that the number of wasps finding each card approximated a Poisson distribution, and proceeded to calculate the expected zero class (cards not found by each type) for each plot.

For the field analyses, the number of trap cards occupied (i.e., the number of trap cards parasitized), the number of offspring for the three different isofemale lines, and the number of lines per trap card were analyzed by fitting linear mixed-effects models, considering weather variation (S1 Table.) and/or estimated number of released individuals as random-effect factors, and the day of release (i.e. age of parasitoids) and experimental isofemale line as fixed-effect quantitative factors. The mixed-effect model is appropriate for this analysis because the weather conditions and the variable initial number of released individuals introduced a variance-covariance structure of the variable responses [44]. When the inter-dependency effects between isofemale lines and day of release was significant, it was included in the model. P-values were obtained by likelihood ratio tests of the full model with the effect in question against the model without the effect in question. Residual plots were checked to confirm no deviation from homoscedasticity or normality. For the analysis we used the ‘lmer’ function from the ‘lmer4’package of the R statistical program [45] to fit the models.

## Results

### Laboratory ranking of the isofemale lines

**Fig 3:**
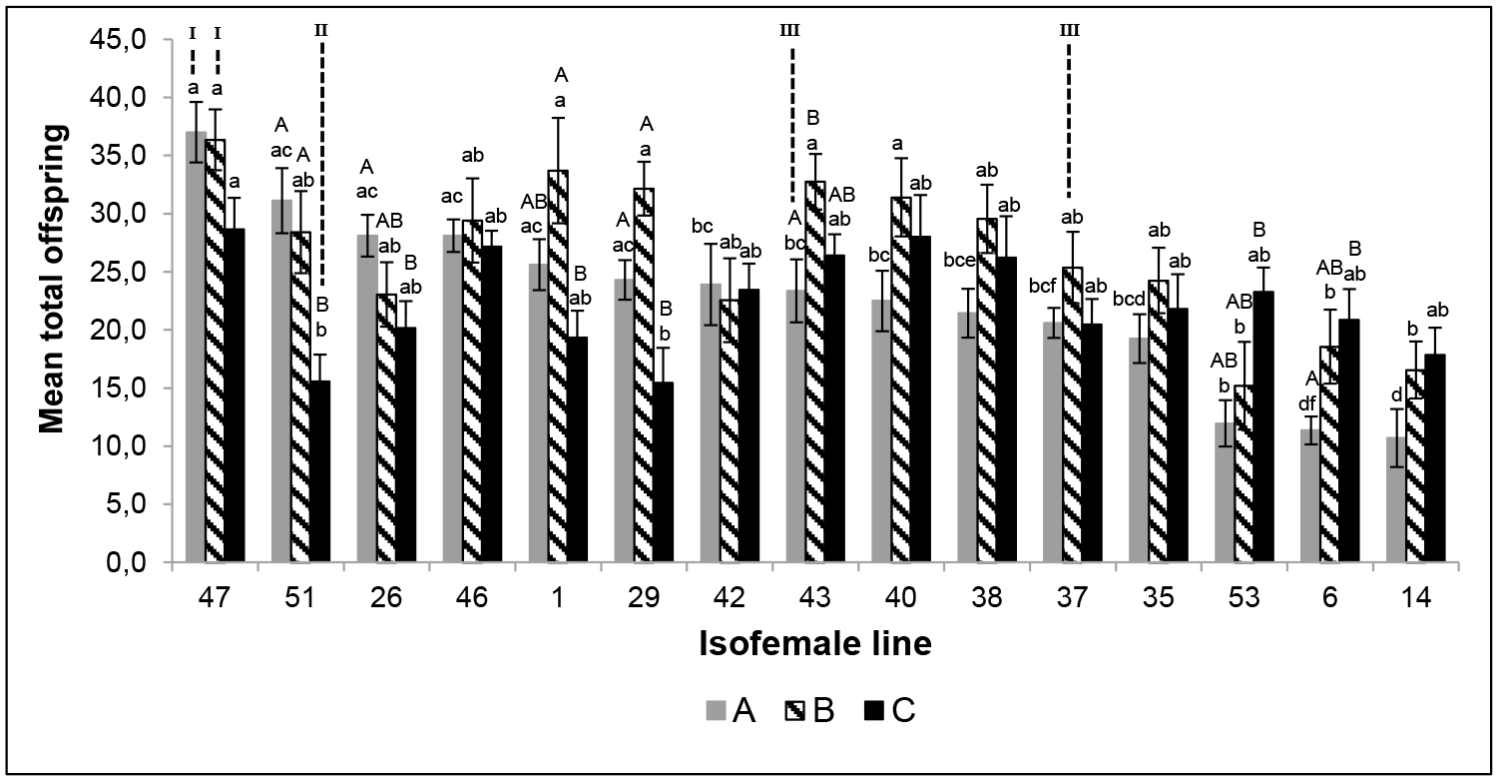
Offspring generated by the different isofemale lines of *Trichogramma pretiosum* on *Ephestia kuehniella* eggs as host. Temperature 25±1°C, RH 60±10%, photophase 14h. Differences among the isofemale lines with the same mitochondrial background, are indicated by lower-case letters above columns with the same shade, as assessed using a Tukey test (p<0.05). Differences among the different mitochondrial backgrounds in each isofemale line are indicated by uppercase letters above the columns, as assessed using a Tukey test (p<0.05). I—Isofemale line classified as the “Best”; II—Isofemale line classified as the “Worst”; III—Isofemale line classified as “Intermediate”.

Under laboratory conditions, the 45 isofemale lines of *T*. *pretiosum* showed clear differences in fertility (measured by the number of offspring generated by each female in three days). There was a main effect of nuclear type, with certain lines generally being more fecund than others (e.g., 47 versus 14 respectively; F_14,872_ = 10.06, p< 0.01; Fig 3). Mitochondrial type was also a significant main effect; lines harboring mitochondrial types A and B were generally more fecund than those with type C (F_2,886_ = 11.36, p< 0.01; Fig 3). However, there was a significant interaction between the two factors (Nuclear type and mitochondrial background), which was evident from the fact that a few nuclear lines actually performed best when carrying mitochondrial type C (e.g., line 14; F_28,844_ = 3.00; p< 0.01; Fig 3).

**Fig 4:**
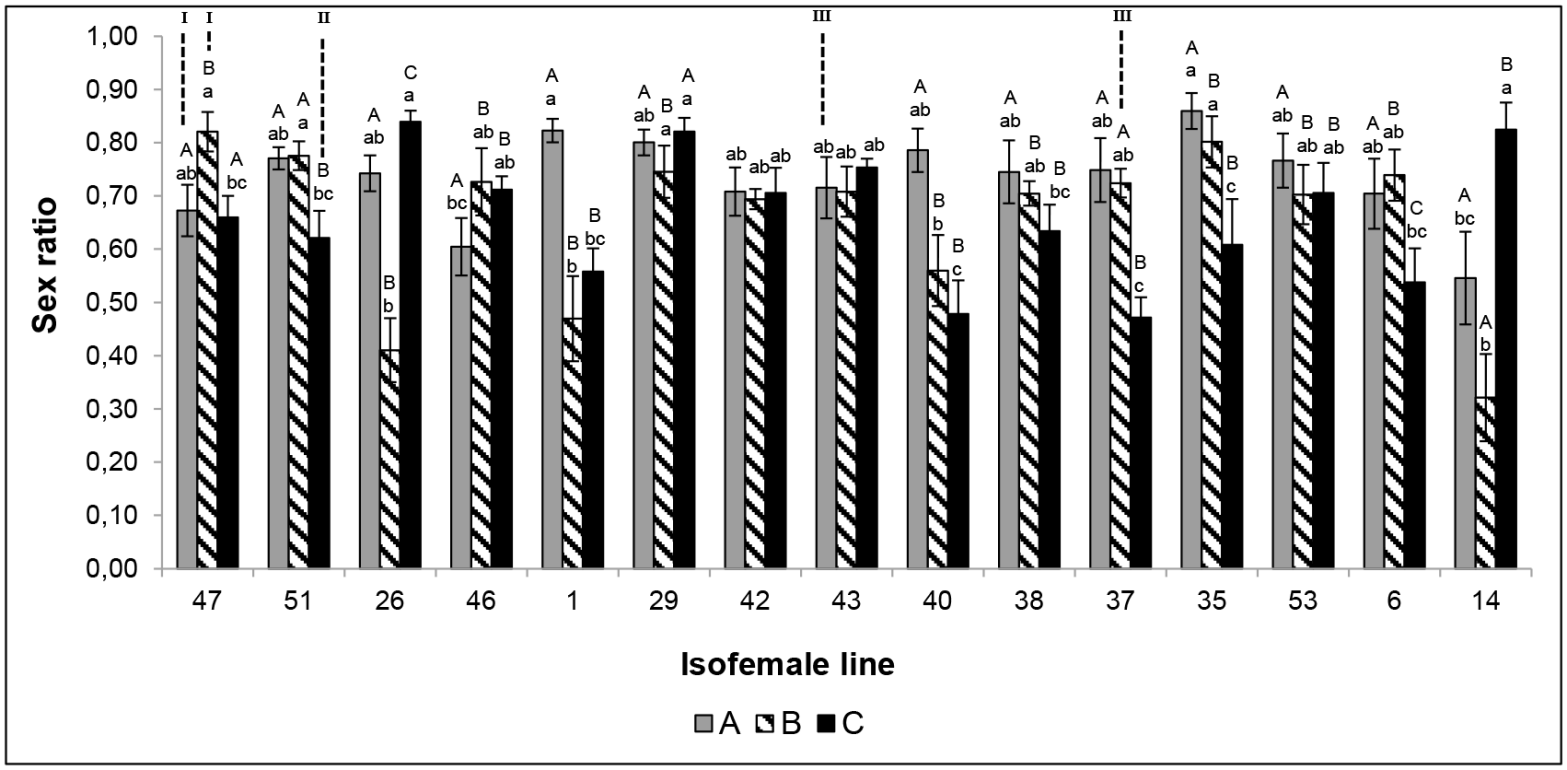
Sex ratio of the different isofemale lines of *Trichogramma pretiosum* on *Ephestia kuehniella* eggs as host. Temperature 25±1°C, RH 60±10%, photophase 14h. Differences among the isofemale lines with the same mitochondrial background, are indicated by lower-case letters above columns with the same shade, as assessed using a Tukey test (p<0.05). Differences among the different mitochondrial backgrounds in each isofemale line are indicated by uppercase letters above the columns, as assessed using a Tukey test (p<0.05). I—Isofemale line classified as the “Best”; II—Isofemale line classified as the “Worst”; III—Isofemale line classified as “Intermediate”.

Both factors also influenced the offspring sex ratio of the different *T*. *pretiosum* isofemale lines. There was a main effect of nuclear type, with certain nuclear lines generally producing broods with higher numbers of females than others (e.g. 35 versus 14 respectively; F_14,872_ = 4.32, p< 0.01; Fig 4). Likewise, mitochondrial type was also a significant main effect, with mitochondrial type A generally resulting in broods with higher numbers of females than types B and C (F_2,886_ = 320.83, p< 0.01; Fig 4). However, the offspring sex ratio was highly dependent on the interaction between the nuclear and mitochondrial backgrounds, which was evident from the fact that a three nuclear lines actually produced the most female-biased broods when carrying mitochondrial type C (e.g., line 14; F_28,844_ = 5.20; p< 0.01; Fig 4).

### Field experiment

Two field releases were carried out, each using a different set of three ranked *T*. *pretiosum* experimental lines. The isofemale lines selected for use in the two field releases are shown in Table 1.

**Table 1:** Ranked lines of *Trichogramma pretiosum* used in the field releases.

Release	Ranked lines		
	Best	Intermediate	Worst
1^st^	47 A	37 B	51 C
2^nd^	47 B	43 A	51 C

A total of 600 trap cards were placed in the field during each release (50 per plot X four plots X three days). Of this total, 291 and 301 cards were parasitized in the first and second releases, respectively.

**Fig 5:**
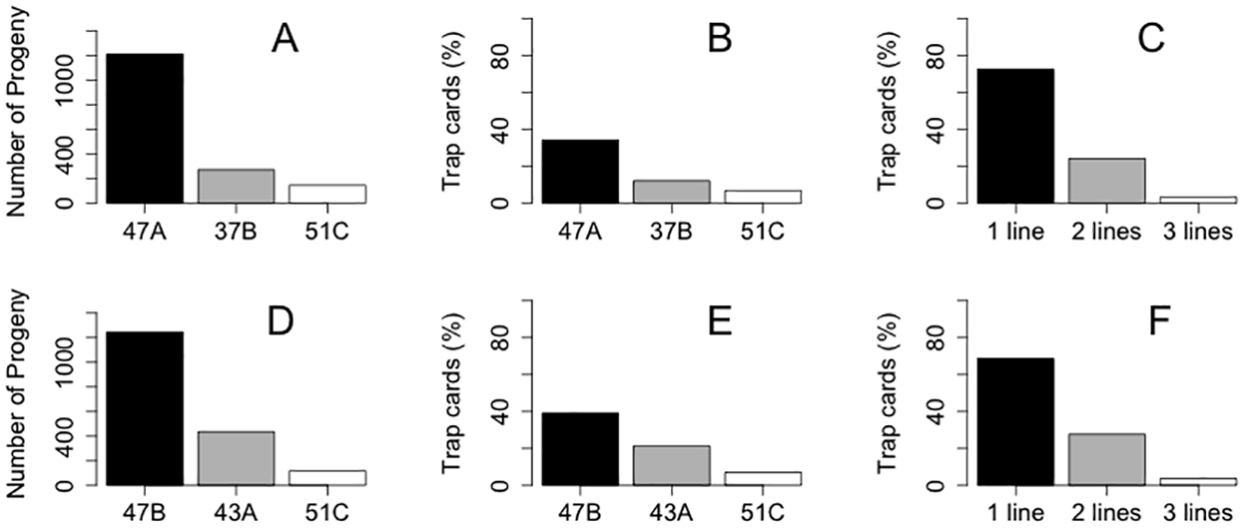
Field success of “Best”, “Intermediate” and “Worst” laboratory-ranked lines of *Trichogramma pretiosum* in two separate releases, measured as: A and D) Number of offspring recovered and analysed by qPCR; B and E) Parasitism of the isofemale lines in the field, measured by the number of trap cards parasitized; and C and F) Co-occurrence (%) of the isofemale lines (measured by the total number of isolines present on the trap cards). A), B) and C) first release; D), E) and F) second release.

The number of wasps recaptured and analyzed through qPCR, that is, the number of offspring from the released wasps was significantly affected by the day of release and isofemale line for the first release (p<0.001, Table 2A). For the second release, was found significance for the interaction between the day of release and isofemale line (p = 0.0025, Table 2A). The interaction term indicates that the number of progeny is explained by the changes in female reproductive performance due to their age (i.e., day of release). The isofemale line ranked as “Best” produced more offspring under field conditions than the other isofemale lines (Fig 5A and 5D). In the first and second releases, the relative density of new emergent individuals corresponded to, 1210 and 1245 from the “Best” line, against 146 and 117 from the “Worst” line (Fig 5A and 5D). This suggests that these lines showed differences of 88 and 91% in the parasitism rate in the first and second releases. It should be taken into account that eight parasitoids were randomly sampled for each trap card, but not all trap cards had at least eight individuals. In the first release, 51% of the trap cards had eight or more wasps, and in the second, 47% had eight or more wasps.

**Table 2:** Estimates of the parameters of the linear mixed models for progeny number, total of trap cards occupied (%) and abundance of lines per trap card for the three different isofemale lines and days after they were released.

	Response	Explanatory factors	Parameter estimate (±SE)
A	Progeny Number	Intercept	102.817 (±17.645)
	(First Release)	Intermediate	-77.422 (±12.863)
		Worst	-87.511 (±12.863)
		2^nd^ day	-3.311 (±12.863)
		3^rd^ day	-4.622 (±12.863)
	Progeny Number	Intercept	120.000 (±7.020)
	(Second Release)	Intermediate	-72.50 (±9.928)
		Worst	-122.50 (±9.928)
		2^nd^ day	-22.75 (±8.512)
		3^rd^ day	-53.00 (±8.512)
		Intermediate × 2^nd^ day	6 (±12.038)
		Worst × 2^nd^ day	28 (±12.038)
		Intermediate × 3^rd^ day	26.5 (±12.038)
		Worst × 3^rd^ day	57.50 (±12.038)
B	Number of trap cards occupied	Intercept	59.00 (±2.904)
	(First Release)	Intermediate	-39.84 (±4.198)
		Worst	-48.00 (±4.107)
		2^nd^ day	-51.50 (±3.631)
		3^rd^ day	-23.00 (±3.631)
		Intermediate × 2^nd^ day	35.00 (±5.135)
		Worst × 2^nd^ day	42.00 (±5.135)
		Intermediate × 3^rd^ day	19.00 (±5.135)
		Worst × 3^rd^ day	19.75 (±5.135)
	Number of trap cards occupied	Intercept	46.00 (±2.992)
	(Second Release)	Intermediate	-19.00 (±4.231)
		Worst	-40.5 (±4.231)
		2^nd^ day	-6.00 (±3.270)
		3^rd^ day	-14.50 (±3.270)
		Intermediate × 2^nd^ day	1.00 (±4.625)
		Worst × 2^nd^ day	10.00 (±4.625)
		Intermediate × 3^rd^ day	2.50 (±4.625)
		Worst × 3^rd^ day	15.00 (±4.625)
C	Number of lines per trap card	Intercept	23.74 (±0.744)
	(First Release)	2 isolines	-14.25 (±1.062)
		3 isolines	-22.50 (±1.062)
		2^nd^ day	-19.494 (±1.22)
		3^rd^ day	-6.738 (±1.22)
		2 isolines × 2^nd^ day	10.75 (±1.502)
		3 isolines × 2^nd^ day	18.25 (±1.502)
		2 isolines × 3^rd^ day	2.00 (±1. 502)
		3 isolines × 3^rd^ day	6.25 (±1. 502)
	Number of lines per trap card	Intercept	16.25 (±1.253)
	(Second Release)	2 isolines	-6.25 (±1.772)
		3 isolines	-15.25 (±1.772)
		2^nd^ day	1.25 (±1.772)
		3^rd^ day	1.50 (±1.772)
		2 isolines × 2^nd^ day	-4.00 (±2.506.)
		3 isolines × 2^nd^ day	-1.00 (±2.506)
		2 isolines × 3^rd^ day	-8.00 (±2.506)
		3 isolines × 3^rd^ day	-2.00 (±2.506)

The number of trap cards occupied by the lines in the first and second releases was also influenced by the changes in the performance of the isofemale lines over the days (significant interaction term between day/isofemale lines, p<0.001 and p = 0.037, respectively). Again, the *T*. *pretiosum* isofemale line that was ranked as the best in the laboratory, found more trap cards than the intermediate-ranked line, which in turn found more than the worst (Fig 5B and 5E; Table 2B). In general the “Best” line of *T*. *pretiosum* occupied more than 40% of the trap cards, while the “Worst” line occupied less than 20%, in both releases.

The occupation of the trap cards by the “Worst” line was influenced by the “Best” and “Intermediate” lines, with a small number of trap cards parasitized simultaneously by the “Worst” and the other two lines. Considering the number of female isolines present on the trap cards, the simultaneous occurrence of the isolines was evaluated. The interaction term between number of isolines present on the trap card and day of release had a significant effect on the abundance of isolines on first and second releases (p<0.001 and p = 0.047, respectively). A high proportion of trap cards were parasitized by only one isofemale line, 72.58% (first release)– 68.6% (second release), and less than 4% of the trap cards were parasitized simultaneously by all three isofemale lines, in both releases (Fig 5C and 5F). The number of trap cards parasitized by two isofemale lines was intermediate in comparison to occupation by one and three isofemale lines, among the days in both releases (Table 2C).

Through the field experiments it became clear that the line classified as the “Best” in laboratory experiments, also performed best in the field (Table 3, Fig 5). The best *T*. *pretiosum* line parasitized more cards than expected in all releases and days, with the exception of day 2 of the first release (Table 3). The experimental line ranked "Best" in the laboratory showed a higher parasitism rate in the field than expected. Numerically, parasitism by the isofemale line ranked as “Best” was highest in both releases, on all days, but the Chi-square analysis did not have an appropriate comparison method to show the differences between the best line and the lines ranked as “Intermediate” or “Worst”.

**Table 3:** Chi-square of expected and observed parasitism of *Trichogramma pretiosum* isofemale lines in field experiment.

					Cards found^4^									Empty cards^5^			Chi-square^6^			Significance^7^		
		Females released^3^			Day 1			Day 2			Day 3			Day			Day			Day		
Release^1^	Plot^2^	47a	37b	51c	47a	37b	51c	47a	37b	51c	47a	37b	51c	1	2	3	1	2	3	1	2	3
First	1	1580	1357	1295	26	9	2	3	3	1	9	8	1	17	39	31	10.1	3.20	4.52	<0.01	0.90	0.02
	2	1668	1517	935	32	11	4	3	1	1	27	9	6	10	41	13	9.4	2.18	5.49	<0.01	0.49	0.02
	3	1596	1253	1007	33	10	8	5	1	1	17	8	4	8	44	24	10.4	3.8	9.5	<0.01	0.28	<0.01
	4	1228	1517	1423	27	7	8	4	0	0	17	5	4	16	43	23	8.4	2.98	8.65	0.0123	0.49	<0.01
		43a	47b	51c	43a	47b	51c	43a	47b	51c	43a	47b	51c									
Second	1	1566	1670	1427	16	22	2	10	16	4	4	13	3	19	18	27	10.6	0.69	5.3	<0.01	0.02	0.03
	2	1557	1688	1430	11	22	3	13	21	4	10	22	4	18	32	21	10.6	0.85	6.76	<0.01	0.02	0.02
	3	1516	1668	1481	16	31	4	14	24	6	13	15	3	34	30	21	9.5	0.78	5.8	<0.01	0.01	0.02
	4	1514	1660	1493	11	17	2	7	19	5	3	13	2	19	37	25	7.9	0.89	5.6	0.01	0.04	0.03

^1^Releases performed in a cornfield in Piracicaba, SP, Brazil

^2^Number of plots used in the field experiment

^3^Number of females released

^4^Number of cards found (parasitized) on each day of the experiment

^5^Number of empty cards, i.e. card not parasitized by any isofemale line on each day of the experiment

^6^Chi-square “modified” calculated

^7^Significance calculated for the Chi-square

The weather conditions varied during the different days of isofemale release (S1 Table). At the end of day one of the first release it rained, and following this rain, the relative humidity rose to 95% and the mean temperature dropped below 20°C. These conditions continued until the third day, when the mean temperature was lower than 23°C and the RH higher than 80%. In the second release, the mean temperature and RH were more stable; the temperature ranged between 25.5 and 26.8°C and the RH from 71 to 75%. A heavy rain fell at the end of day 3.

## Discussion

Genetically divergent isofemale lines of *T*. *pretiosum* showed differences in their reproductive performance in the laboratory, similar to observations previously reported for *T*. *pretiosum* lines that were reared under similar conditions [10, 19, 29, 30, 46, 47]. Along with parasitization, seven other traits vary due to genetic variation in *Trichogramma* species, namely: i) walking behavior; ii) spatial distribution; iii) handling time; iv) superparasitism; v) sex ratio; vi) rate of development; and vii) preimaginal viability [48]. The present study used isofemale lines that had been reared in the laboratory for five years. Each isoline was practically homozygous, so that each genotype was unable to adapt to laboratory conditions since no genetic variation was present.

The lack of genetic variability is not an obstacle for the success of biological control, completely homozygous lines of *Encarsia formosa* Gahan, a 100% homozygous, thelytokous species, has been used since the 1930s to control whitefly in Europe [49]. However, loss of variability can occur at the beginning of laboratory rearing, due individuals better adapted to the artificial rearing conditions have advantages over others, therefore leading to elimination of some alleles and domestication [50, 51, 52]. These losses can be avoided dividing the founding population into subpopulations [29, 30]. Once will be expected to lose an different set of alleles during their establishment and prolonged laboratory rearing, but the original variation can be largely restored by mixing the subpopulations shortly before they are released in the field [14, 53, 54, 55]. For the same isofemales lines used in the present experiment, was assessed that the mixing 26 isolines for two generations presented a reproductive fitness higher than a (pure) isoline with high reproductive fitness [29, 30]. The literature suggest that to maintain and restore 95% of the common alleles, present in the original field population, hybridizing at least 25 inbred lines, when working with haplodiploid insect parasitoids [29, 30, 55, 56].

Our results demonstrated that *T*. *pretiosum* has the capacity to parasitize and persist (at least for three days) under field conditions in Brazil, which are quite different from southern California, USA, where they were initially collected. The effectiveness of *Trichogramma* in the field largely depends on their searching behavior (habitat location, host location) and host preference (recognition, acceptance, suitability) [4]. In our experiments *T*. *pretiosum* was reared on *E*. *kuehniella* eggs, and both experiments used these factitious eggs as a model host. More-pronounced differences among lines of *T*. *carverae* were found when natural hosts were used as a “trap card” in vineyard field experiments [19]. Our data showed very robust differences among the lines, even using the factitious host. Parasitism was recorded throughout the plot, even on the most distant trap cards, although the size of the plots was chosen considering that *T*. *pretiosum* has a maximum dispersal area of approx. 81 m^2^ [38], and the area in our experiments was 100 m^2^. Overall, weather conditions in the field during our experiments were similar to the artificial climate under which the insects were maintained in the laboratory; temperature 25°C, RH 60%. However, on day two of the first release, the temperature dropped below 20°C and the relative humidity was higher than 95%. As a result, *T*. *pretiosum* activity in the field practically stopped. The relative humidity is low during most of the year in southern California, approximately 60% [57], and this difference in climate conditions may have affected our *T*. *pretiosum* isofemale lines. Temperatures between 15 and 20°C are a threshold for the field efficacy of different *Trichogramma* species [58, 59, 60]. This information is complemented with one experiment that showed a direct correlation between solar radiation and temperatures < 15°C on the activity of *T*. *pretiosum* and *T*. *evanescens* Westwood in the field [60], while a negative correlation between RH and parasitism was described [58]. Less than 4% of the individuals of *Trichogramma minutum* Riley took flight at 20°C, while a much higher percentage flew at 25°C [61].

Our experiments showed a relationship between the laboratory performance and field performance of the *T*. *pretiosum* lines. In both releases, the parasitism capacity in the field showed the same pattern as in the laboratory, i.e. the line ranked as the “Best” in the laboratory also performed best in the field. The laboratory measure of fertility is a useful predictor for *T*. *minutum* performance in the field [58]. A similar correlation between laboratory and field performance in *T*. *brassicae* Bezdenko was described, but also other parameters were considered to be even better predictors of field performance [59]. In a fourth *Trichogramma* species, *T*. *carverae*, laboratory fertility was not a good predictor of field performance [19]. In contrast to the previous experiment performed by [58, 59], in our experiment, the direct competition between the lines may have influenced the results. In the present research, all *T*. *pretiosum* lines originated from a different environment from where they were released.

The direct competition between the lines may explain the large differences that we found in the field. One experiment showed that isofemale lines have different capacities to search for the host and walk on leaf surfaces [62], and the authors suggested that the area searched is an important parameter that should be related to the efficiency of biological control agents. Considering this, future experiments might evaluate if the lines ranked as “Best” in the laboratory show a high capacity for host search and walking on the leaf.

In our results, less than 4% of the cards were parasitized by more than three isofemale lines. This may be related to the detection of marking pheromones left behind by ovipositing females, to avoid hyperparasitism of already parasitized eggs [63]. The low parasitism and low fertility rates recorded in the field for the “Worst” isofemale lines may also be influenced by marking pheromones, since the “Best” lines are likely to find the resource patches (trap cards) more quickly.

Mitochondrial DNA as a marker was a rapid and efficient technique. Considering the small size of *T*. *pretiosum* individuals, the use of another kind of marker might result in less accurate field experiments. A marker should not affect the behavior of the insects, it must be durable, and the manipulation and release of insects cannot affect their life-span and behavior [64]. Not all these assumptions could be proven through our experiment, due to the loss of experimental lines, which led to a change in the experimental design. The expected “neutrality” of the mitochondrial background did not occur in our laboratory experiments, since significant interactions arose. Nevertheless, the real effect of the mitochondrial background is not clear and remains to be explored for *T*. *pretiosum*, since our field experiments did not test the interactions between the mitochondrial types released simultaneously.

The use of the different mitochondrial haplotypes was not toxic to the insect and environment, was relatively easily to apply (after the populations were “created”), and the lines were clearly identifiable, which satisfies almost all assumptions for an ideal marker [65]. Unfortunately, the instrumentation cost is high, but once a qPCR machine is available, the cost of the reagents to perform a mitochondrial marker assay is not prohibitively expensive.

Considering that the molecular backgrounds affect the biology of the insects, the use of mitochondrial DNA as a marker should take into account previous biological data (i.e. laboratory performance) to avoid error in any biological trials using this method. It should be considered that the mitochondrial DNA influences the “fitness” of the parasitoids, i.e. each line that receives a specific mitochondrial background will have specific biological requirements, which may differ from the requirements of the same “nuclear line” with a different mitochondrial background. In addition to the possibility of using mitochondrial DNA as a marker, our data also showed that the reproductive performance index in laboratory conditions was a strong predictor for the field success of mass-reared *T*. *pretiosum* isofemale lines.

## Supporting Information

S1 Table Weather data from the experimental area, during the first and second releases. Source: Biosystems Engineering Department, ESALQ/USP.
(TIF)
