# Supplementary figures and images for: Laboratory Performance Predicts the Success of Field Releases in Inbred Lines of the Egg Parasitoid Trichogramma pretiosum (Hymenoptera: Trichogrammatidae)
Source: PLoS One. 2016 Jan 5;11(1):e0146153. doi: 10.1371/journal.pone.0146153 (PMC4701434; doi:10.1371/journal.pone.0146153)

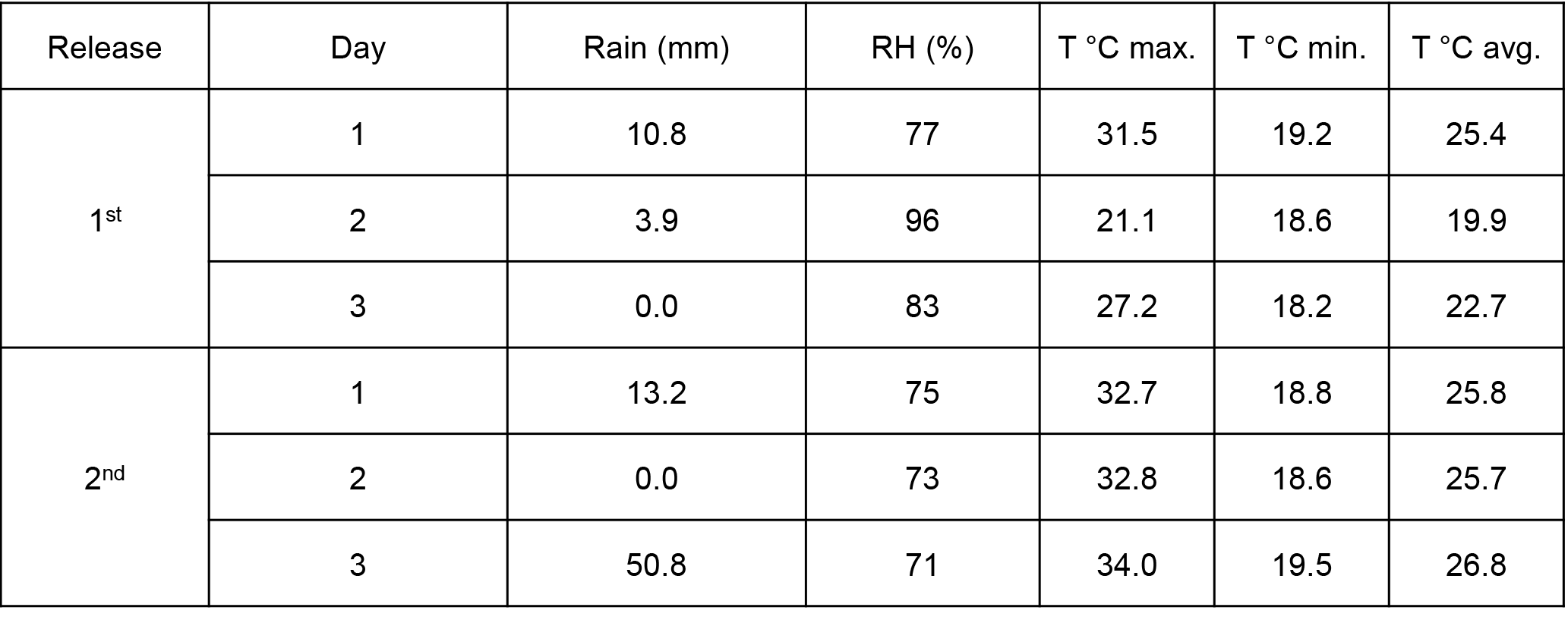

Supplement: S1 Table — Source: Biosystems Engineering Department, ESALQ/USP. (TIF) [file pone.0146153.s001.tif]
